# Is the intraoperative air leak test effective in the prevention of colorectal anastomotic leakage? A systematic review and meta-analysis

**DOI:** 10.1007/s00384-016-2616-4

**Published:** 2016-06-13

**Authors:** Zhouqiao Wu, Remondus C. J. van de Haar, Cloë L. Sparreboom, Geesien S. A. Boersema, Ziyu Li, Jiafu Ji, Johannes Jeekel, Johan F. Lange

**Affiliations:** Department of Gastrointestinal Surgery, Peking University Cancer Hospital & Institute, No. 52, Fu-Cheng Road, Hai-Dian District, Beijing, 100142 China; Department of Surgery, Erasmus University Medical Center, Rotterdam, The Netherlands; Department of Neuroscience, Erasmus University Medical Center, Rotterdam, The Netherlands; Department of Surgery, Havenziekenhuis, Rotterdam, The Netherlands

**Keywords:** Anastomotic leakage, Colorectal surgery, Air leak test, Prevention

## Abstract

**Objective:**

The intra-operative air leak test (ALT) is a common intraoperative test used to identify mechanically insufficient anastomosis. This meta-analysis aims to determine whether ALT aids to the reduction of postoperative colorectal anastomotic leakage (CAL).

**Methods:**

A literature search was performed to select studies in acknowledged databases. Full text articles targeting ALT during colorectal surgery were included. Quality assessment, risk of bias, and the level-of-evidence of the inclusions were evaluated. ALT methodology, ALT(+) (i.e., leak observed during the test) rate, and postoperative CAL rate of the included studies were subsequently analyzed.

**Results:**

Twenty studies were included for analysis, in which we found substantial risks of bias. A lower CAL rate was observed in patients who underwent ALT than those did not; however, the difference was not significant (*p* = 0.15). The intraoperative ALT(+) rate greatly varied among the included studies from 1.5 to 24.7 %. ALT(+) patients possessed a significantly higher CAL rate than the ALT(−) patients (11.4 vs. 4.2 %, *p* < 0.001).

**Conclusions:**

Based on the available evidence, performing an ALT with the reported methodology has not significantly reduced the clinical CAL rate but remains necessary due to a higher risk of CAL in ALT(+) cases. Unfortunately, additional repairs under current methods may not effectively decrease this risk. Results of this review urge a standardization of ALT methodology and effective methods to repair ALT(+) anastomoses.

## Introduction

Colorectal anastomotic leakage (CAL) is one of the most dangerous short-term complications after colorectal surgery, attributing to one third of postoperative mortality [1]. To prevent CAL, substantial efforts have been made. Among them, the air leak test (ALT) is apparently the most frequently performed intraoperative test to identify a mechanically insufficient anastomosis [2]. Typically, certain countermeasures such as additional sutures or a temporary protective stoma construction are performed when a leak (e.g., leakage of air bubbles or dye) was observed during the test.

Though being performed by a majority of colorectal surgeons, it remains inconclusive whether performing ALT and the immediate repair of the ALT(+) cases (i.e., leak of air/dye observed in the test) is beneficial in preventing CAL. This may be due to at least two variables: first, the etiology of CAL is multi-factorial, comprised of communication between intra- and extra-luminal content (e.g., suture dehiscence), anastomotic infection (e.g., peritonitis) and healing disturbances (e.g., ischemia). Performing an ALT may provide limited assistance in detecting CAL due to causes other than anastomotic mechanical failure. Second, various ALT techniques with different outcomes have been reported, which increases the concern that whether such varying techniques may cause different results in detecting mechanically failed anastomosis and eventually lead to different clinical intervention and outcomes. To date, no systematic review or meta-analysis is available to provide solid evidence to support a routine ALT application. To this end, we performed this review aiming to determine whether implementing the intraoperative air leak test might aid to reduce CAL.

## Method

### Literature search strategy

The literature search for this systematic review was performed in January 2015 according to the PRISMA (Preferred Items for Reporting of Systematic Reviews and Meta-Analyses) guidelines in databases including Medline, Embase, Cochrane, Web-of-Science, and Google Scholar databases. No restrictions regarding publication date or language have been applied during the search. We restricted our search to human studies. The following search strategy was used in Embase and modified in other databases accordingly: ((*air NEAR*/*3* (leak* OR pressure* OR insufflat* OR burst* OR tight* OR compress* OR inject* OR deflat*) *NEAR*/3 test*) *OR* ((((air OR leak* *OR pressure*) *NEAR*/3 test*):*ab*,*ti*) *AND* (“*anastomosis leakage*”/*exp OR* “*intestine anastomosis*”/*exp OR anastomosis*/*exp OR* (anastomo* OR leak*):*ab*,*ti*))) *AND* (“*intestine surgery*”/*exp OR intestine*/*exp OR* “*large intestine disease*”/*exp OR* (intestin* OR colorect* OR colon* OR rectum OR rectal OR bowel* OR sigmoidectom* OR hemicolectom* *OR anorectal OR anal OR anus*):*ab*,*ti*).

### Study selection

Titles and abstracts of identified articles were independently screened by two authors (R.H., C.S.) for relevance to the subject. Full text articles were included if they targeted intraoperative air leak test during colorectal surgery and reported the CAL rate accordingly. Reviews, letters to editors, congress, and meeting abstracts were excluded. Hereafter, the references of the selected articles were screened for any relevant articles.

### Quality assessment and data extraction

Quality assessment and risk of bias were reviewed by two independent authors (R.H., C.S.) according to The Cochrane Collaboration’s tool for assessing risk of bias [3]. The tool assesses the risk of bias and applicability concerns by means of six key domains including sequence generation, allocation concealment, blinding, incomplete outcome data, selective outcome reporting, and other sources of bias. Afterwards, the Level of Evidence was evaluated according to the Levels of Evidence (LOE) 2011 from the Centre for Evidence Based Medicine, Oxford [4].

The definition of CAL and the method of performing the air leak test of the included articles were recorded. The clinical endpoints, postoperative clinical or radiological colorectal manifestations of CAL were included for analysis. We assessed ALT performance, intraoperative leakage rate, and the corresponding CAL rates. We also evaluated several subgroups including the analysis of CAL rate in ALT (+) vs. ALT(−) groups. ALT(+) is defined as leak of air/dye observed in the test; ALT(−) is defined as leak of air/dye not observed in the test.

### Statistical analysis

Our primary objective was to determine whether performing ALT reduces CAL. We made a comparison between CAL rates in patients who underwent ALT vs. CAL rates in patients that did not undergo the test. We also compared the CAL rate in the ALT(+) patients vs. the ALT(−) ones to determine whether ALT(+) patients have a higher CAL incidence after surgery. For pooling data and calculating a pooled mean for each outcome, the Mantel–Haenszel method was applied using a random-effect model; mean differences with a 95 % confidence interval were calculated. Statistical heterogeneity was assessed using *Q* statistic and *I*^2^ statistics.

### Sensitivity analysis

To assess the individual effect of the studies on the overall outcome, a sensitivity analysis was performed. One study was removed at a time to determine whether this would influence the significance of the pooled outcome.

Analyses were performed using Review Manager software (RevMan version 5.3; The Nordic Cochrane Centre, Copenhagen, Denmark).

## Results

### Literature search results, level of evidence, and risk of bias

**Fig. 1:**
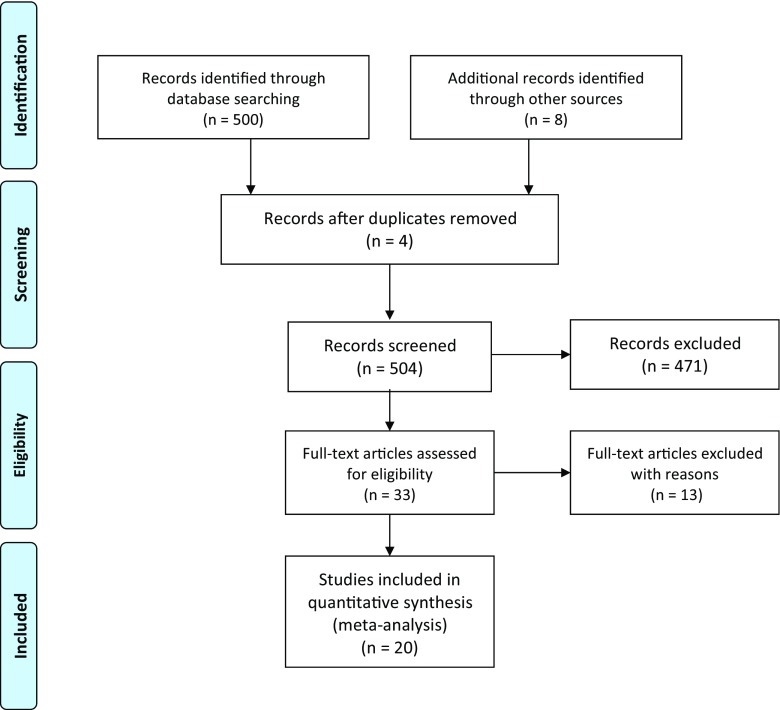
Flow chart of the literature search according to the PRISMA guideline

In total, there were 500 studies identified during the systematic search, of which, 12 studies appeared to be relevant to the study question and were therefore included for analysis. An additional 8 articles were selected from the references, boosting the total to 20 included articles (Fig. 1). In total, 5283 patients were included for analysis, with 2395 of them undergoing ALT. The inclusions contained 2 randomized trials, 7 cohort studies, and 11 case series (Table 1).

**Table 1:** Overview of the included studies

Author	No. of patients	Study design	LOE	AL definition	Method of air leak test	Consequence of ALT+
Lazorthes and Chiotassol [18]	82	Prospective case series	4	(1) Clinical signs (not defined) (2) Routinely radiological control (enema)	Air insufflation into the rectum using a catheter with the anastomosis under irrigation of saline	Reinforcing sutures or defunctioning colostoma
Davies et al. [5]	33	Prospective case series	4	(1) Clinical signs: mild (pyrexia, ileus) vs. major (need for colostomy, fistula) (2) Routinely radiological control (enema)	Air insufflation into the rectum using a catheter with the anastomosis under irrigation of saline.	Reinforcement sutures and retested
Beard et al. [19]	143	RCT	1b	(1) Clinical signs (not defined) (2) Routinely radiological control (enema)	Air insufflation into the rectum using an endoscope with the pelvis under irrigation of saline	Oversewing and retested
Griffith et al. [6]	60	Prospective case series	4	Clinical signs: (1) fecal fistula (2) Anastomotic breakdown seen at laparotomy or post-mortem examination in association with peritonitis (3) Clinical features of AL confirmed by sigmoidoscopy or rectal examination	Air insufflation into the rectum using a catheter with the anastomosis under irrigation of saline	Further stitches until satisfactory ALT
Pritchard et al. [7]	82	Prospective case series	4	Clinical signs (prolonged ileus, unexplained fever, tenderness and distention or rectal discharge). Confirmed by Gastrografin enema. Diagnosis was based on radiography	Air insufflation into the rectum using a catheter with the anastomosis under irrigation of saline	Defunctioning stoma
Yalin et al. [16]	21	Prospective case series	4	Clinical signs (not defined)	Air insufflation into the rectum using a catheter with the anastomosis under irrigation of saline	Additional sutures or diverting stoma
Sakanoue et al. [17]	70	Prospective cohort	2b	Clinical signs (not defined)	Air insufflation into the rectum using an endoscope with the anastomosis under irrigation of saline	Reinforcing sutures and diverting stoma
Vignali et al. [8]	55	Prospective case series	4	(1) Clinical signs: fecal fistula to the wound, to the drain tract, or to the vagina or pelvic sepsis supported by radiologic evidence of leak (2) Routinely radiological control	Insufflation of air into the bowel with the anastomosis under irrigation of saline	Temporary stoma
Schmidt et al. [9]	788	Retrospective cohort	2b	Clinical signs: gas, pus, or fecal discharge from a drain, pelvic abscess, peritonitis, discharge of pus per rectum or rectovaginal fistula	Air insufflation into the rectum using an endoscope with the anastomosis under irrigation of saline	Protective stoma
Ishihara et al. [20]	73	Prospective case series	4	Not described	Insufflation of air into the bowel with the anastomosis under irrigation of saline	Additional sutures or redoing of the anastomosis
Lanthaler et al. [21]	122	Prospective cohort	2b	Not described	Insufflation of air into the rectum with the anastomosis under irrigation of saline	Oversewn, protecting loop ileostomy or Hartmann procedure
Ricciardi et al. [10]	998	Retrospective cohort	2b	Clinical signs: the presence of luminal contents through a drain or wound site or abscess cavity causing inflammation (i.e., fever, leukocytosis, or fecal discharge).	Insufflation of air through a proctoscope or flexible endoscope with the anastomosis under irrigation of saline	(1) Repair without diversion or (2) unplanned proximal diversion or (3) takedown of anastomosis with new anastomotic construction and no diversion
Li et al. [11]	244	Retrospective cohort	2b	Clinical signs: peritonitis, feculent substances or gas from the drain, and sepsis or the presence of abscess with demonstrable anastomotic leak by clinical, endoscopic, or radiologic examination	Transanal insufflation of air using an endoscope with the anastomosis under irrigation of saline	Additional interrupted sutures
Shamiyeh et al. [22]	338	Retrospective cohort	2b	Not described	400 cc air insufflation into the colon using a syringe with the anastomosis under irrigation of saline	Oversewn or redoing anastomosis
Ivanov et al. [12]	60	RCT	1b	Clinical signs: gas leakage, pus, or fecal discharge from the drain, clinical picture of anastomotic dehiscence (increased body temperature, stomach painful at palpation, auscultatory evidence of absent peristalsis, signs of liquid–gas levels at abdominal x-ray, leukocytosis) with or without a confirmation by sigmoidoscope, and the presence of intra-abdominal abscess verified either by ultrasonography or abdominal CT.	Air insufflation into the rectum using a sigmoidoscope with the anastomosis under irrigation of saline	Repair by single layer extramucosal sutures
Lieto et al. [13]	124	Prospective cohort	2b	Clinical signs: presence of signs of peritonitis or abdominal sepsis with or without evidence of luminal content and/or gas through the drain, and demonstrable anastomotic breakdown by endoscopic and/or radiologic examination	Insufflation of air using an endoscope with the anastomosis under irrigation of saline	Oversewn with interrupted sutures
Kamal et al. [23]	415	Retrospective case series	4	Not described	Air insufflation during sigmoidoscopy	Revision of the anastomosis
Kim et al. [24]	363	Retrospective case series	4	Not described	Not described	Re-resection and re-doing anastomosis with or without diverting stoma, suturing at disrupted site with or without diverting stoma, or only diverting stoma were performed by surgeon’s preference
Xiao et al. [14]	198	Prospective case series	4	Clinical signs: (1) Peritonitis and related abnormalities: pelvic or perineal pain or tenderness, tachycardia, fever, and increased white blood cell (WBC) count (2) Gas, fecal, or purulent discharge from the pelvic drain, drain tract, or anus (3) Pelvic abscess or fluid collection (4) Rectovaginal fistula. The diagnosis was verified by clinical, endoscopic, radiologic investigations, or laparotomy	Air insufflation into the rectum using an rectoscope with the anastomosis under irrigation of saline	Repair at the discretion of the operating surgeon, either with additional sutures or redoing anastomosis
Vignali et al. [15]	1014	Retrospective case series	4	Clinical signs: fecal fistula from a wound, drain tract, or vagina; confirmed by sigmoidoscopy or rectal examination; or pelvic sepsis supported by radiologic evidence of leak	Insufflation of air into the bowel with the anastomosis under irrigation of saline	Reinforcing sutures, if sutures were not possible a defunctioning stoma was formed

The risk of bias of each inclusion was evaluated and listed in Table 2. Substantial risks of bias were observed among different studies, mostly focusing on the lack of randomization and clear definition of CAL.

**Table 2:** Risk of Bias of the included studies

Author	Sequence generation	Allocation concealment	Blinding	Incomplete outcome data	Selective outcome reporting	Other sources of bias
Lazorthes and Chiotassol [18]	−	−	−	+	+	+
Davies et al. [5]	−	−	−	+	+	+
Beard et al. [19]	+	+	?	+	+	+
Griffith et al. [6]	−	−	−	+	+	+
Pritchard et al. [7]	−	−	−	+	+	+
Yalin et al. [16]	−	−	−	+	?	+
Sakanoue et al. [17]	−	−	−	−	?	−
Vignali et al. [15]	−	−	−	+	+	+
Vignali et al. [8]	−	−	−	+	+	+
Schmidt et al. [9]	−	−	−	+	+	−
Ishihara et al. [20]	−	−	−	+	?	+
Lanthaler et al. [21]	−	−	−	+	+	+
Ricciardi et al. [10]	−	−	−	+	+	+
Li et al. [11]	−	−	−	+	+	+
Shamiyeh et al. [22]	−	−	−	+	+	+
Ivanov et al. [12]	+	?	?	+	+	+
Xiao et al. [14]	−	−	−	+	+	+
Lieto et al. [13]	−	−	−	+	+	+
Kamal et al. [23]	−	−	−	+	+	+
Kim et al. [24]	−	−	−	+	+	+

### CAL definition, ALT methodology

Among the inclusions, only 11 studies [5–15] provided detailed diagnostic criteria for CAL. Five studies [8–10, 16, 17] diagnosed CAL based on clinical manifestations, while eight studies [5, 7, 8, 12–14, 18, 19] provided both clinical and radiological diagnostic criteria of CAL. There were five studies [20–24] that did not provide any references with regard to the diagnosis of CAL.

Various methods of ALT tests were used in the included studies. As listed in Table 1, we found that with the exception of one study [24]. Despite the fact that all other studies reported their methods of ALT evaluation, the methods themselves varied greatly between studies. Not all studies reported the volume of the inflated gas/dye, while the reported volume varied from 60 mL [6] to 400 mL [22]. No study mentioned intraluminal pressure measurements during ALT. When a leak was observed during ALT, i.e., ALT(+), different repair methods were applied varying from reinforcing sutures up to reconstruction of the anastomosis or performing a diverting stoma [17] (Table 1).

### Clinical CAL rate in ALT patients vs. non-ALT patients

**Fig. 2:**
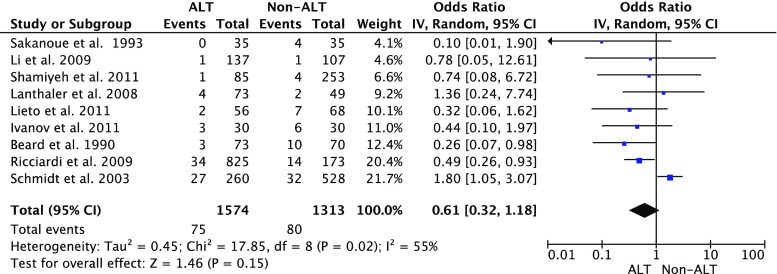
Clinical colorectal anastomotic leakage rate in air leak test (ALT) patients vs. non-ALT patients

As is shown in Fig. 2, nine studies reported a comparison of the clinical CAL rate between the patients with ALT and those without ALT. Although a lower CAL rate was found in the patients with ALT, no significant difference was found when compared to the patients without ALT (*P* = 0.15). The heterogeneity among the studies was significant (*P* = 0.02, *I*^2^ = 0.55).

**Fig. 3:**
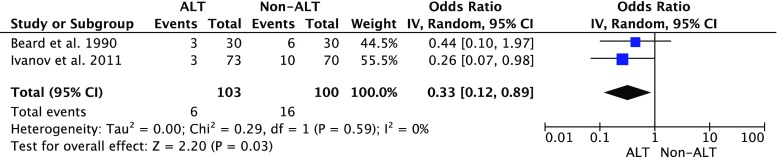
Clinical colorectal anastomotic leakage rate in air leak test (ALT) patients vs. non-ALT patients: subgroup analysis LOE 1b. *LOE* level of evidence

**Fig. 4:**
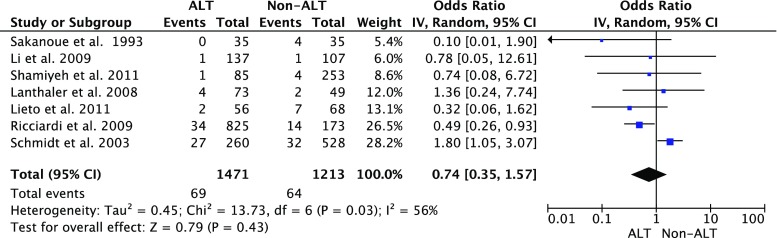
Clinical colorectal anastomotic leakage rate in air leak test (ALT) patients vs. non-ALT patients: subgroup analysis LOE 2b. *LOE* level of evidence

Subgroup analysis showed that combining the data of the LOE 1b studies [12, 19] showed a significant difference in the CAL rate between patients with ALT and those without ALT (Fig. 3), while such difference was not significant in the LOE 2b studies (Fig. 4). The combined CAL rate in the patients with ALT remained stable at 5.8, 4.7, and 4.9 % in the LOE1b, 2b, and 4, respectively. On the contrary, the CAL rate in the patients without ALT was reported as 16 % in the LOE 1b studies, which was higher than the rate of 5.3 % in the LOE 2b trials.

### CAL rate in ALT(+) vs. ALT(−)

**Fig. 5:**
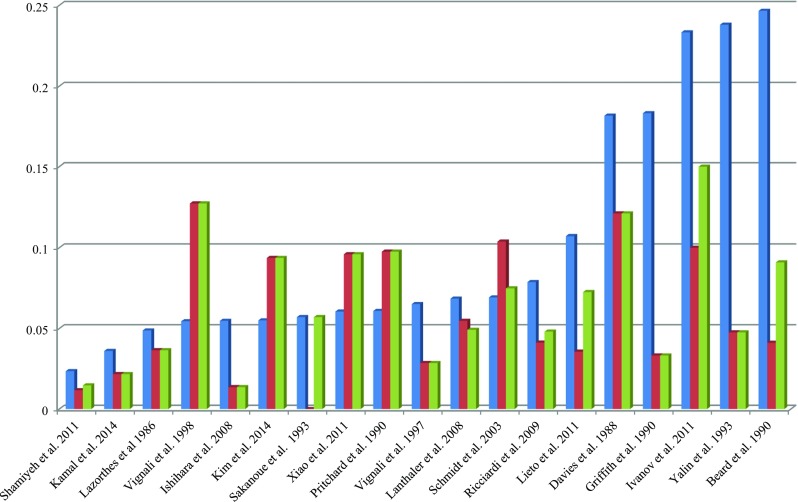
Intraoperative ALT (+) rate, postoperative CAL rate in ALT (+) cases, and overall postoperative CAL rate. *Bars* in *blue* indicate the intraoperative positive rate of the air leak test, i.e., ALT(+) rate; *bars* in *red* indicate the postoperative CAL rate in the ALT(+) patients; *bars* in *green* indicate the overall postoperative CAL rate in all the included patients in each study respectively. *CAL* colorectal anastomotic leakage, *ALT* air leak test, *ALT(+)* indicates that leak was observed during the test

**Fig. 6:**
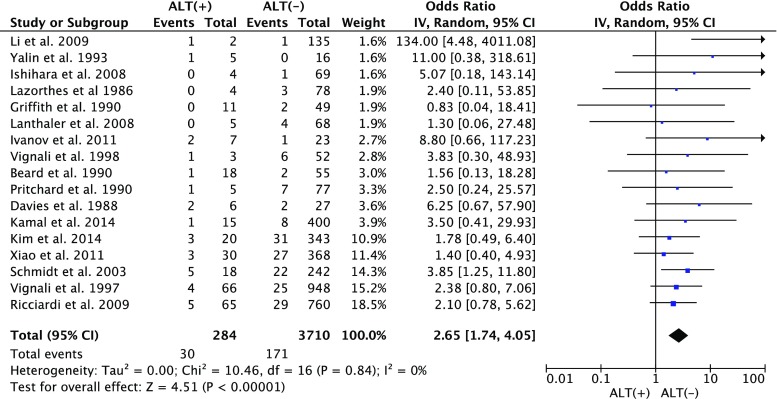
Colorectal anastomotic leakage rate in ALT(+) patients vs. in ALT(−) patients. *CAL* colorectal anastomotic leakage, *ALT* air leak test, *ALT* air leak test, *ALT(+)* indicates that leak was observed during the test, *ALT(−)* indicates that no leak was observed during the test

As is shown in Fig. 5, the intraoperative positive rate of ALT varies among different studies (1.5 to 24.7 %). While the clinical CAL rate in those intraoperative ALT(+) patients was 11.4 %, compared to 4.2 % in ALT(−) patients. The meta-analysis showed a significant difference (*P* < 0.001) between these two groups (Fig. 6), with no significant heterogeneity between studies (*P* = 0.84, *I*^2^ = 0).

### Sensitivity analysis

Except from one study, exclusion of the others had no influence on outcome significance. Exclusion of one heavily weighted article from Schmidt et al. [9], however, resulted in a major change in significance in the clinical CAL rate in ALT patients vs. non-ALT patients evaluation. With this article included, an OR of 0.61 [0.32, 1.19] (*P* = 0.15) was found. After exclusion, an OR of 0.46 [0.29, 0.74] (*P* = 0.001) was calculated.

## Discussion

Air leak test (ALT) is the most frequent performed intraoperative test to detect mechanically insufficient colorectal anastomoses for intraoperative repair. This meta-analysis summarizes the clinical evidence regarding the application of ALT. We found that with current evidence, performing ALT has not significantly reduced the clinical CAL rate after surgery, but it remains necessary due to a significantly higher risk of CAL in patients with a positive leak during the test. The standardization of ALT in future studies is urgently needed to further verify the effectiveness of ALT and its future applications.

As is shown from our data, no significant reduction of CAL rate is seen in the meta-analysis of the CAL rates between patients who underwent ALT and those who did not. Although subgroup analysis showed a significant difference in the RCT (LOE1b) studies, the limited numbers of patients and the extraordinarily high CAL rate in the patients without ALT increase the concern with regards to the reliability of the difference. Particularly, since neither of the two LOE1b studies blinded the surgeons during postoperative investigation, the observer bias may influence the diagnosis of CAL after surgery. For future studies, it is important to ensure double blinding when performing a RCT on such topic.

In the sensitivity analysis, the primary comparison in this meta-analysis was heavily influenced by one study with a large number of patients [9]. Exclusion of that study resulted in a significant outcome in favor of ALT application. This substantially influenced the statistical analysis and the corresponding *p* value. Moreover, it increased the uncertainty of the actual ALT effectiveness. However, we chose to include this study in the final analysis because the reporting bias was considered to be limited in the LOE 2b studies since during operation surgeons were not aware of the comparison between patients underwent ALT vs. those who did not. Of course, one possible bias in LOE 2b studies is the selection bias: surgeons may only subject anastomoses that are likely to leak to ALT but not the firm ones, which seems also explain the similar CAL rate between patients with and without ALT. This bias does exist in many of our LOE 2b inclusions, but not in the studies from Shamiyeh et al. [22] and Schmidt et al. [9], which compared historical data (without routine ALT) to recent data (with routine ALT). Our further analysis found similar results when we ruled out the selected ALT cases (data not shown).

According to the current data, whether performing ALT significantly reduces the CAL rate after surgery is, at best, inconclusive. The abovementioned limitations, together with other factors including the heterogeneity in ALT methodology and outcome measurements, might all contribute to the inconclusive results. However, such results undoubtedly sound the call for a worldwide standardization of the air leak test.

A direct explanation of our data might be that ALT is not useful in the prevention of CAL and may thus be abandoned. We, probably together with all surgeons, certainly oppose such explanation because any colorectal surgeon would have seen a mechanically failed anastomosis (e.g., anastomotic dehiscence) detected by ALT, in which the avoidance of a ALT would certainly cause catastrophic CAL. Rather than the superficial interpretation, our results have shown one clear cause of the inconclusive effect of ALT: the significantly higher CAL rate in ALT(+) patients demonstrates that ALT(+) patients are still under higher risk of developing CAL even though a repair procedure was performed in most cases. We recognize that it is certainly reasonable to assume that ALT(+) may have an even higher CAL rate without the repairing procedures. However, since ALT simply detects the mechanical insufficiency, our data at least demonstrates that the current repairing strategies in the ALT(+) cases, varying from additional sutures to performing a diverting stoma, have not effectively eliminated the mechanical risks of CAL in those positive cases. The high CAL rate in ALT(+) patients may extensively attenuate the preventive effect of ALT, resulting in the similar CAL rate between patients with and without ALT.

Though having been performed for decades, no standardized methodology or consensus has been reached, which is confirmed by our results. The fact that one inflated 60 mL of air during the test while another injected 400 mL of saline is disturbing and raises the question whether we are performing the same ALT. Unfortunately, the results from our study are not encouraging in this regard. Despite the lack of detailed methods, intraoperative ALT resulted in a positive rate varying from 1.5 to 24.7 % among different studies [11, 19]^.^ Considering that intraoperative repair was applied in most ALT(+) patients, we should be aware that such a diverse range of positive rate strongly implies the possibility of overtreatment in many patients, particularly in centers with a high rate of ALT(+) cases. We intended to further explore whether there is any difference among the intraoperative repairing methods in reducing postoperative CAL rate, while unfortunately such analysis was not possible with the current data since it requires much detailed information that are not reported in most inclusions.

From a biomechanical point of view, a standardized volume of the injected air or water is difficult to establish because of the variation in patients’ anatomy. Thereby, pressure should be considered as a means for standardization. It is important to note that an anastomosis (either handsewn or stapled) may not sustain intraluminal pressure as high as one may imagine. Although systematic evaluation of the burst pressure is not yet available, studies report that a newly constructed colorectal anastomosis bursts at the pressure around 70 to 184 mmHg [25]. Compared to this pressure, injecting 400 mL of saline seems dangerous if not properly controlled. Therefore, a pressure indicator might be suggested during ALT. Actually, measuring the intra-luminal pressure has been included as a very standard method in measuring the early-stage anastomotic strength in animal studies [26, 27]. Although such technique is not presently available for human patients, we believe it is urgently needed. A barometer can be combined then with endoscopy or certain inflating devices to ensure a safety and ease of application.

## Conclusion

In conclusion, currently available evidence regarding the value of ALT in prevention of CAL contains substantial risks of bias. Based on the evidence, performing ALT with the reported methodology has not effectively reduced the clinical CAL rate after surgery. This is partly because a positive result in ALT still predicts a higher risk of postoperative CAL, and additional repairs with current methods do not decrease this risk. However, the evidence also suggests that performing ALT is necessary to identify patients with a higher risk of CAL. Being the quality control step of colorectal anastomosis, the air leak test is in dire need for worldwide standardization. Future studies with a higher level of evidence (e.g., double blinded RCT) should be initiated to verify the effectiveness of ALT.
